# Optimal combination of arsenic trioxide and copper ions to prevent autoimmunity in a murine HOCl-induced model of systemic sclerosis

**DOI:** 10.3389/fimmu.2023.1149869

**Published:** 2023-03-30

**Authors:** Charlotte Chêne, Dominique Rongvaux-Gaïda, Marine Thomas, François Rieger, Carole Nicco, Frédéric Batteux

**Affiliations:** ^1^ INSERM U1016, Institut Cochin, Département 3I “Infection, Immunité Et Inflammation”, Université Paris Cité, Paris, France; ^2^ R&D Department, MEDSENIC SAS, Strasbourg, France; ^3^ Service d’immunologie Biologique, AP-HP-Centre Université Paris Cité, Hôpital Cochin, Université Paris Cité, Faculté De Médecine, Paris, France

**Keywords:** arsenic trioxide, ATO, copper, fenton-like reaction, fibrosis, systemic sclerosis, Nrf2, ROS

## Abstract

**Introduction:**

Systemic sclerosis (SSc) is a rare chronic autoimmune disease characterized by diffuse fibrosis of the skin and internal organs and vascular abnormalities. The etiology and physiopathology are complex due to the heterogeneity of its overall clinical presentation. Arsenic trioxide (ATO) has been proven to be effective against SSc, sclerodermatous Graft-versus-Host Disease, multiple sclerosis, Crohn’s disease or systemic lupus erythematosus animal models and has demonstrated promising effects in human clinical trials. Its efficacy was shown to be related at least in part to the generation of Reactive Oxygen Species (ROS) and the selective deletion of activated immune cells and fibroblasts. However, ATO can induce some adverse effects that must be considered, especially when used for the treatment of a chronic disease.

**Methods:**

We evaluate here, *in vitro* and in a mouse model of SSc, the improved efficacy of ATO when associated with a Fenton-like divalent cation, namely copper chloride (CuCl_2_), also known to trigger the production of ROS.

**Results:**

In preliminary experiments *in vitro*, ATO 1 µM + CuCl_2_ 0.5 µM increased ROS production and increased apoptosis of NIH 3T3 murine fibroblasts compared to 1 µM ATO alone. *In vivo*, in the HOCl-induced mouse model of SSc, co-treatment with ATO 2.5 μg/g + CuCl_2_ 0.5 μg/g significantly alleviated clinical signs such as the thickening of the skin (p<0.01) and cutaneous fibrosis, in a manner equivalent to treatment with ATO 5 µg/g. Our results provide evidence that co-treatment with ATO 2.5 μg/g + CuCl_2_ 0.5 μg/g decreases the number of B cells and the activation of CD4^+^ T lymphocytes. The co-treatment substantially blocks the NRF2 signaling pathway, increases H2O2 production and results in the improvement of the health status of mice with experimental SSc.

**Conclusion:**

In conclusion, copper combined with ATO treatment halved the concentration of ATO needed to obtain the same effect as a high dose of ATO alone for the treatment of SSc mice. The strategy of using lower doses of drugs with different mechanisms of action in combination has many potential advantages, the first being to lessen the potential side effects induced by ATO, a drug with side effects quickly increased with dosage.

## Graphical Abstract

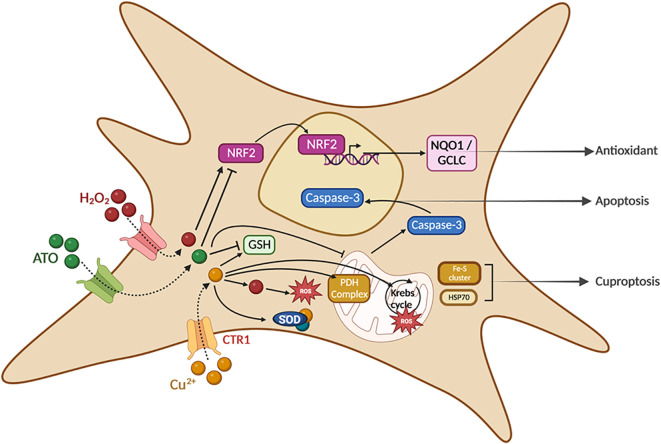

## Introduction

Systemic sclerosis is a complex autoimmune disease characterized by autoimmunity, vasculopathy and progressive connective tissue fibrosis, particularly of the skin and visceral organs (1, 2). SSc causes extensive systemic tissue damage and death occurs as the result of end‐stage organ failure. Among connective autoimmune and inflammatory diseases, SSc is the one with the highest mortality rate per number of cases. Numerous studies suggest that reactive oxygen species (ROS) play an important role in the pathogenesis and development of this disease in both mouse models and humans (3, 4). In SSc, ROS are continuously produced by diseased fibroblasts, leading to their proliferation and the production of collagen and extensive fibrosis (5). Arsenic trioxide (ATO) has features of a pro-oxidant chemotherapeutic agent that increases ROS production, consumption of glutathione (GSH), intracellular oxidoreductase interference and reduction of thioredoxin activity (6). In a previous study, we showed that ATO decreases HOCl-induced skin and lung fibrosis in a HOCl-induced SSc mouse model (7). These beneficial effects were shown to be accompanied by ROS generation that adds its effects to the constitutive production of ROS by SSc fibroblasts, resulting in the selective killing of diseased fibroblasts containing low levels of GSH. ATO is currently used to treat APL, but repeated daily high doses of ATO over several weeks have been reported to induce adverse effects on liver, heart and peripheral nerves. Although these side effects are reversible to some extent, their occurrence often leads to the interruption or even the cessation of ATO therapy (8). Moreover, SSc is a chronic connective tissue disease and its treatment needs to take into account the effect of, and potentially avoid, excessively high, toxic cumulative doses. As increasing ROS in diseased fibroblasts has been shown to be effective for the treatment of SSc with ATO, we sought to find a way of associating with ATO a drug that could further increase ROS production in diseased fibroblasts but without the documented side effects of ATO. The well-known Fenton chemistry is the oxidation process activated by Fe(II) salts in the presence of H_2_O_2_ that generates radical species in solution and oxidizes a wide range of organic substrates with high activity (9). A Fenton-like reaction is based on the same principle with other metals (10). The production of ROS by these reactions is known to induce a strong oxidative stress in activated cells leading to their apoptotic death (11–13). We have previously tested several divalent cations (copper, iron, manganese, gold, zinc) for their ability to potentiate *in vitro* the pro-oxidative effect of ATO on human and murine cancer cells (14). Among them Cu^2+^ (in its CuCl_2_ molecular form) showed the most significant effects on the induction of oxidative stress and apoptosis of cancer cells when used along with ATO. In the present study, we associated CuCl_2_ and ATO in *in vitro* experiments on activated mouse fibroblasts, which are key players in the pathophysiological processes of SSc, and we also tested this combination *in vivo* in a mouse model of HOCl-induced SSc.

## Materials and methods

### Chemicals

CuCl_2_ (GMP grade batches) was obtained from ChemCon (Freiburg, Germany) and ATO (Arscimed, clinical grade batch, stock solution at 1 mg/mL) was obtained from MEDSENIC (Strasbourg, France).

### Cell lines

NIH 3T3, a mouse embryonic fibroblast cell line, was purchased from ATCC (ATCC CRL-1648). Cells were maintained throughout the experiments in a 75-cm^2^ flask (Falcon 250 mL 75 cm^2^, #353135) in Dulbecco’s Modified Eagle Medium (Sigma-Aldrich, Saint-Quentin- Fallavier, France) containing 10% fetal bovine serum (FBS) (Gibco, USA), 1% penicillin-streptomycin (Gibco, USA), 1% ciprofloxacin (Fresenius Kabi, France) and 1% fungizone (Gibco, USA).

### Cell cultures

Cells were seeded in 96-well flat-bottom plates (Falcon, Corning, #353077) and incubated for 48 hours under different experimental conditions at 37°C and 5% CO_2_. Three plates were prepared in parallel to measure H_2_O_2_ production and cell viability.

### Measurement of H_2_O_2_ production

Fibroblasts were coated in triplicate in 96-well microplates (2.10^4^ cells/well). After 48 hours of treatment with ATO ± CuCl_2_, the supernatant was removed and 50 µL per well of 50 µg/mL 2’, 7’-dichlorodihydrofluorescein di-acetate (H_2_DCF-DA; Sigma-Aldrich, Saint-Quentin-Fallavier, France) in PBS was added.

The H_2_O_2_ production was assessed by spectrofluorimetry using a fusion spectrofluorometer (Packard). Fluorescence intensity was recorded immediately (T0 hours) and after 6 hours of incubation (T6 hours). Fluorescence excitation/emission maxima were, for H_2_DCF-DA, 485/530 nm.

### Cell viability

The medium was removed and cells were stained with 0.5% crystal violet and 30% ethanol in PBS for 30 minutes at room temperature. After two PBS washes, ethanol was added to the pellet and absorbance was measured at 550 nm using a fusion spectrofluorometer (15).

### Animals

Six-week-old female BALB/c mice (H2^d^) were purchased from Janvier laboratory (Le Genest-Saint-Isle, France) and maintained with food and water ad libitum. The animals were given humane care, in accordance with the guidelines of our institution (Université Paris Cité, Paris). The protocols and all experimental procedures of this study were approved by the Ethics Committee of Paris Cité University (Animal facilities C75-14-05, DAP #26065).

### Experimental procedure for induction of SSc

HOCl was produced extemporaneously by adding 140 μL of NaClO (9.6% as active chorine) to 18 mL of KH_2_PO_4_ solution (100 mM; pH 7.2). HOCl was controlled by spectrophotometry at 292 nm and then adjusted to obtain an optical density between 0.7 and 0.9 as previously described (5).

Mice were randomly distributed into experimental (n=10 per group) and control groups (n=5). HOCl (200 μL) was injected with a 27-gauge needle intradermally into the shaved backs of the mice, 5 days a week for 6 weeks. Only 5 mice were used in the control group to reduce the number of animals and follow the 3R rule, as we know from previous experiments that this number is sufficient for statistically significant results in this SSc model (SSc mice). PBS-control mice received the same pattern of injections with 200 μL of PBS.

### Treatment of SSc mice

We used this well-established mouse model of SSc, triggered by intradermal injections of HOCl (7). SSc mice were randomized and treated with intraperitoneal injections of ATO ± CuCl_2_ or vehicle (PBS) for 36 days. Mice were divided into five groups.

*In vivo* the treatment by ATO 5 µg/g conditions was selected according to literature data and to our previous results in cGvHD and SSc mice models. In order to reduce the cumulative dose of injected ATO, CuCl_2_ was used as co-treatment with two times decreased dose of ATO (2.5 µg/g). Preliminary tests showed that a high dose of CuCl_2_ (4 µg/g) alone is toxic for mice (data not shown). Finally, a low dose of CuCl_2_, equivalent to doses used in food supplements, potentiated the effect of a low dose of ATO (2.5 µg/g) to the same level as that of a high dose of ATO (5 µg/g), as studied in a mouse model of cGvHD (14). Therefore, we used the CuCl_2_ 0.5 µg/g dosage.

The HOCl-SSc-control mice received PBS and the four remaining groups received a daily injection of ATO alone 2.5 µg/g or 5 µg/g or ATO 2.5 µg/g with CuCl_2_ 0.5 µg/g or CuCl_2_ alone 0.5 µg/g, 5 days a week during 6 weeks.

### Assessment of fibrosis

Skin thickness of the shaved backs of the mice was measured every week with a calliper (expressed in millimeters). These histogram results shown in **Figure 2B** correspond to day 35 measurements.

### Reverse transcription - quantitative PCR (RT-qPCR)

Total mRNA was extracted from crushed samples using trizol reagent (ThermoFisher Scientific, USA, #15596026) according to the manufacturer’s protocol. The expression level of *α-SMA*, *Collagen I*, *NRF2, NQO1*, *GCLC* and *CCL22* were evaluated by RT-qPCR in the skin. The QuantiTect SYBR^®^ Green RT-qPCR Kit on a LightCycler 480 II instrument (Roche Applied Science, France) was used to perform one-step RT-qPCR. It was carried out for 45 cycles, with a denaturing phase of 15 seconds at 94°C, an annealing phase of 30 seconds at 60°C and an extension of 30 seconds at 72°C. Samples were normalized to mRNA expression of housekeeping genes (*β-actin*) and results were expressed as fold increase using the formula 2−^ΔΔCt^. Primers used for PCR are listed in **Supplementary Table 1**.

### Isolation of spleen cells

For each mouse, spleen cell suspensions were prepared after hypotonic lysis of erythrocytes in potassium acetate solution (ACK: NH_4_Cl 0.15 M + KHCO_3_ 1 mM + Na_2_EDTA 0.1 mM) followed by three washes in complete RPMI medium with 10% heat-inactivated FBS, 1% streptomycin-penicillin, 1% ciprofloxacin, 1% fungizone and 1% sodium pyruvate.

### Flow cytometry analysis of mouse spleen cell subsets

Splenocytes were prepared as described above. Cells were incubated with the appropriate labelled antibody at 4°C for 30 minutes in PBS with 2% FBS. Flow cytometry was performed on a FACS Fortessa II flow cytometer (BD Biosciences) according to standard techniques. For the characterization of splenocytes, the monoclonal antibodies used were: CD3-AF700 (17A2, #100216), CD4-BV510 (GK1.5, #100449), CD25-PE (PC61.5, #12-0251-81), B220-Pacific Blue (RA3-6B2, #103230), CD69-PE DAZZLE (H1.2F3, #104536), CD11b-PerCP Cy5.5 (M1/70, #101228), F4/80-BV711 (BM8, #123147), CD86-BV510 (GL-1, #105039), from eBiosciences (ThermoFisher Scientific, France). Data were analyzed with FlowJo software (Tree Star, Ashland, OR, USA).

### ELISA cytokine detection

Spleen cell suspensions (2.10^6^ cells/well) were seeded in 12-well plates and incubated for 3 hours for the adhesion of macrophages; then the supernatant containing the lymphocytes was collected and placed in another 12-well plate. Macrophages and lymphocytes were cultured with complete RPMI medium with 10% heat-inactivated FBS, 1% streptomycin-penicillin, 1% sodium pyruvate, 1% ciprofloxacin and 1% fungizone. Macrophages were stimulated for 48 hours with a final concentration of 100 ng/mL LPS (from *E. coli* serotype 0127: B8 from Sigma Aldrich #L319) and lymphocytes for 72 hours with a final concentration of 5 µg/mL of concanavalin A (Sigma-Aldrich, Saint-Quentin Fallavier, France). The supernatant was collected and stored at -80°C.

Assessment of IL-17A, IL-6 and TGF-β was performed using specific mouse ELISA kits from eBiosciences (Invitrogen - ThermoFisher Scientific, France). Concentrations were calculated from a standard curve according to the manufacturer’s protocol.

### Statistical analysis

The results were analyzed with GraphPad Prism8. A one-way test ANOVA with Bonferroni’s correction was used to determine the differences between two experimental groups. A difference with p<0.05 was considered as significant. All of the quantitative data are expressed as mean ± standard error of the mean (SEM).

## Results

### Effects of ATO 1 μM + CuCl_2_ on ROS production and viability of NIH 3T3 cells

**Figure 1:**
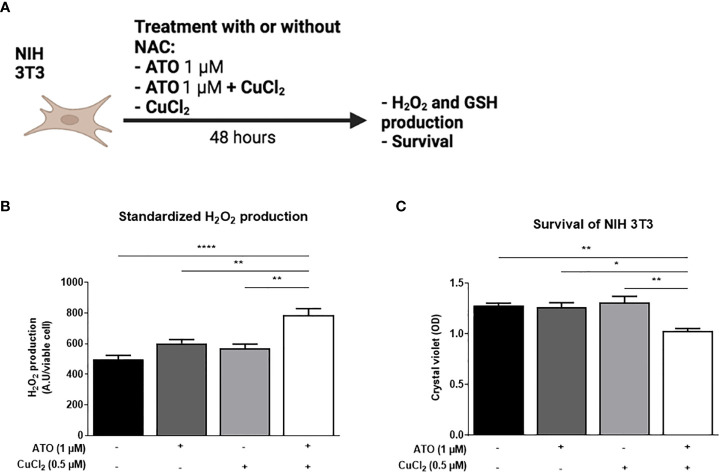
Pro-oxidant effect of ATO 1 μM + CuCl_2_ on NIH 3T3 cells. **(A)** Schematic representation of the *in vitro* experiment on NIH 3T3 cells. The cells were treated for 48 hours with ATO at 1 μM +/- CuCl_2_. The H_2_O_2_ and survival of NIH 3T3 cells were measured by absorbance. **(B)** Standardized H_2_O_2_ level produced by NIH 3T3 cells in culture after treatment during 48 hours with one concentration of ATO (1 µM) and with CuCl_2_ (0.5 µM). H_2_DCF-DA was measured by spectrofluorometry and the results (AU) were divided by cell viability established by colorimetry (crystal violet). **(C)** Cell viability of NIH 3T3 cells in culture measured after treatment during 48 hours with one concentration of ATO (1 µM) and with CuCl_2_ (0.5 µM). NS: not significant; *p<0.05; **p<0.01; ****p<0.0001. The results are the mean of 3 replicates per sample.

We measured the effects of ATO (1 µM) with or without CuCl_2_ (0.5 µM) on the production of H_2_O_2_ by NIH 3T3 cells and on cell viability (**Figure 1A**). We observed an increase in H_2_O_2_ production of 59% (p<0.0001) when the cells were treated with ATO (1 µM) and CuCl_2_ compared to untreated cells and also to cells treated with ATO alone or CuCl_2_ alone at the same dosage (p<0.01; **Figure 1B**). Concerning cell viability, the co-treatment of cells with CuCl_2_ and ATO decreased cellular viability by 20% compared to untreated cells (p<0.01). CuCl_2_ in combination with ATO treatment potentiated the effect of ATO alone or CuCl_2_ alone at the same dosage (p<0.05 and p<0.01, respectively; **Figure 1C**).

### Effects of ATO 2.5 μg/g + CuCl_2_ on the development of cutaneous fibrosis in HOCl-induced SSc mice

**Figure 2:**
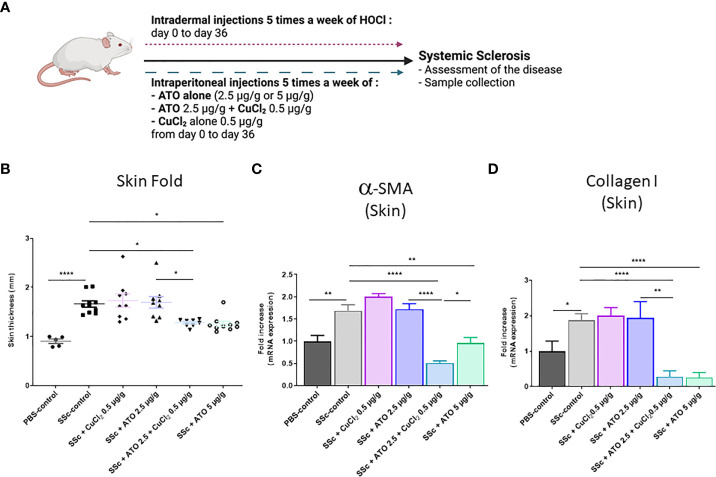
Effects of ATO 2.5 μg/g + CuCl_2_ on skin fibrosis. **(A)** Schematic representation of the experimental induction of SSc in mice. The mice received 5 injections of HOCl per week for 6 weeks intradermally as well as the various treatments intraperitoneally. Mice were monitored weekly for weight and clinical signs of SSc for 36 days. **(B)** Evolution of skin-fold thickness in millimeters at day 35. **(C, D)** *α-SMA* and *Collagen I* mRNA expression in the skin. Data are presented as 2^(−ΔΔCT)^ relative to the levels of *β-actin*. *p<0.05; **p<0.01; ****p<0.0001. The results for each group are the mean of the measurements obtained per mouse: PBS-control (n = 5); SSc-control (n = 10); SSc-CuCl_2_ 0.5 µg/g (n = 9, because 1 mouse died); SSc-ATO 2.5 µg/g (n = 9); SSc-ATO 2.5 µg/g + CuCl_2_ 0.5 µg/g (n = 10); SSc-ATO 5 µg/g (n = 10). *Ex vivo* measurements were realized in duplicate for each mouse.

As expected, repeated injections of HOCl in BALB/c mice (HOCl-SSc mice) (**Figure 2A**) induced a 73% increase in skin thickness at day 35 compared to the PBS-control group (**Figure 2B**; p<0.0001). The co-treatment with ATO 2.5 μg/g + CuCl_2_ significantly decreased skin thickness by 23% compared to the SSc-control group (p<0.05). This effect was equivalent to that resulting from the treatment of animals with a high dose of ATO (5 μg/g), which decreased skin thickness by 24% compared to the SSc-control group (p<0.05). However, the addition of CuCl_2_ to a low dose of ATO (2.5 µg/g) was significantly more effective to decrease dermal thickness than the same low dose of ATO alone (p<0.05 **Figure 2B**). Expression of the two pro-fibrotic markers*α-SMA* and *Collagen I* was increased by 69% and 87%, respectively, in the HOCl-SSc- mice compared to the PBS-control group (p<0.01 and p<0.05, respectively). Treatment with ATO 2.5 μg/g + CuCl_2_ decreased by 70% the expression of *α-SMA* (p<0.0001) and by 85% the expression of *collagen I* (p<0.0001), compared to the HOCl-SSc-mice. A high dose of ATO alone decreased by 43% the expression of *α-SMA* (p<0.01) and by 86% the expression of *collagen I* (p<0.0001) compared to the HOCl-SSc-mice. No significant differences were observed between HOCl-SSc mice treated with PBS, with CuCl_2_ alone and with low dose ATO alone (**Figures 2C, D**).

### ATO 2.5 μg/g + CuCl_2_ acts on the NRF2 signaling pathway

**Figure 3:**
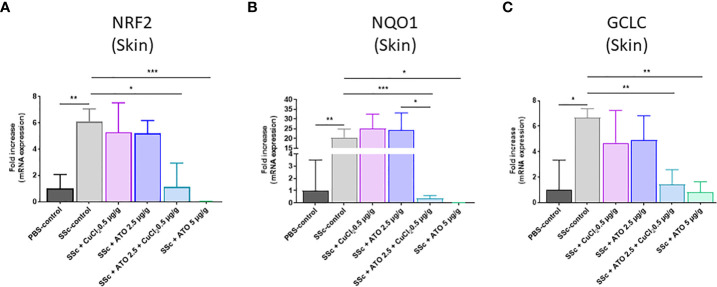
Mechanism of action of ATO 2.5 μg/g + CuCl_2_. **(A–C)** *NRF2*, *NQO1* and *GCLC* mRNA expression in the skin. Data are presented as 2^(−ΔΔCT)^ relative to the levels of *β-actin*. *p<0.05; **p<0.01; ***p<0.001. The results for each group are the mean of the measurements obtained per mouse: PBS-control (n = 5); SSc-control (n = 10); SSc-CuCl_2_ 0.5 µg/g (n = 9); SSc-ATO 2.5 µg/g (n = 9); SSc-ATO 2.5 µg/g + CuCl_2_ 0.5 µg/g (n = 10); SSc-ATO 5 µg/g (n = 10). *Ex vivo* measurements were realized in duplicate for each mouse.

*NRF2* is highly expressed in the diseased HOCl-SSc-mice compared to the PBS-control group (relative expression in PBS-control group = 1 and relative expression in HOCl-SSc-control group = 6.0699; p<0.05). The co-treatment and treatment with high dose ATO decreased the expression of *NRF2* by 81% and 99.6%, respectively (p<0.01) (**Figure 3A**). We also assessed the effect of these treatments on *NQO1* and *GCLC*, two target genes of the NRF2 pathway. The expression of the two target genes was increased in the HOCl-SSc-control group compared to the PBS-control group: p<0.01 for *NQO1* (relative expression in PBS-control group = 1 and relative expression in HOCl-SSc-control group = 20.5954) and p<0.05 for GCLC (relative expression in PBS-control group = 1 and relative expression in HOCl-SSc-control group = 6.6559). Co-treatment with ATO+Cu^2+^ and treatment with high dose of ATO significantly reduced both *NQO1* and *GCLC* gene expression compared to the HOCl-SSc-mice, by 98% and 99%, respectively, for *NQO1* and by 79% and 88%, respectively, for *GCLC* (p<0.001 and p<0.05, respectively, for *NQO1*; p<0.01 and p<0.01, respectively, for *GCLC*). CuCl_2_ potentiated the effect of low dose ATO, which reached an equivalent level to that observed with a high dose of ATO (**Figures 3B, C**).

### Effect of ATO 2.5 μg/g + CuCl_2_ on immune cell number and activation

**Figure 4:**
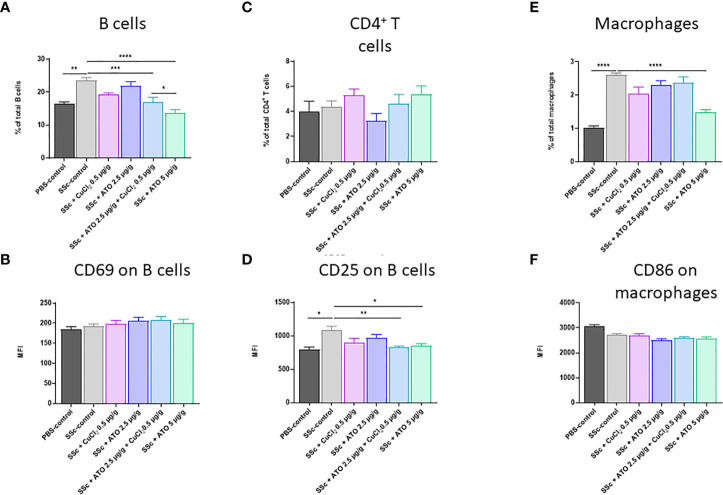
Effects of ATO 2.5 μg/g + CuCl_2_ on innate and adaptive immune cells. **(A–D)** Flow cytometry of splenic B and CD4^+^ T cells. Splenic B cells were gated on CD3^-^ B220^+^. Splenic CD4^+^ T cells were gated on CD3^+^ CD4^+^ positive cells. **(E, F)** Flow cytometry of splenic macrophages. Macrophages were gated on F4/80^+^ CD11b^+^. *p <0.05; **p<0.01; ***p<0.001; ****p<0.0001. The results for each group are the mean of the measurements obtained per mouse: PBS-control (n = 5); SSc-control (n = 10); SSc-CuCl_2_ 0.5 µg/g (n = 9); SSc-ATO 2.5 µg/g (n = 9); SSc-ATO 2.5 µg/g + CuCl_2_ 0.5 µg/g (n = 10); SSc-ATO 5 µg/g (n = 10).

On the day of sacrifice, we evaluated the effect of the co-treatment on the number of immune cells and on their activation status using flow cytometry. We observed an increase of 44% in the percentage of total B lymphocytes in the HOCl-SSc-mice compared to the PBS-control group (p<0.05). The co-treatment with ATO 2.5 μg/g + CuCl_2_ significantly decreased the B lymphocytes count by 28% (p<0.001) compared to the HOCl-SSc-control group. High dose ATO (5 μg/g) decreased the B lymphocyte count by 42% (p<0.0001) compared to the HOCl-SSc-control group (**Figure 4A**). However, despite the decrease in the B lymphocyte count induced by the co-treatment with ATO+CuCl_2_, we did not observe any difference in B cell activation status between experimental groups (**Figure 4B**).

Furthermore, we did not observe any modification in the percentage of CD4^+^ T lymphocytes in the different experimental groups (**Figure 4C**). Interestingly, the induction of SSc by HOCl was followed by an increase of 36% in CD4^+^ T lymphocyte activation (CD25^+^) compared to the PBS-control group (p<0.05). ATO 2.5 μg/g + CuCl_2_ significantly decreased CD25 levels on CD4^+^ T lymphocytes by 23% (p<0.01) compared to the HOCl-SSc-mice and reached the level observed in the PBS-control group and in the HOCl-SSc group treated with high dose ATO 5 μg/g (p<0.05) (**Figure 4D**). High dose ATO decreased the CD25 levels on CD4^+^ T lymphocytes by 21% (p<0.05) compared to the HOCl-SSc-control group.

Finally, the percentage of macrophages in the spleen was higher in the HOCl-SSc-control group than in the control group (p<0.0001) (**Figure 4E**). We found that the co-treatment had no influence on the number of macrophages. However, a 43% decrease in the number of macrophages was observed in the group treated with high-dose ATO (5 μg/g) compared to the HOCl-SSc-control group (p<0.0001) (**Figure 4E**). We did not observe any differences in macrophage activation status regardless of the experimental group considered (**Figure 4F**).

### The effect of ATO 2.5 μg/g + CuCl_2_ on lymphocyte and macrophage cytokine secretion

**Figure 5:**
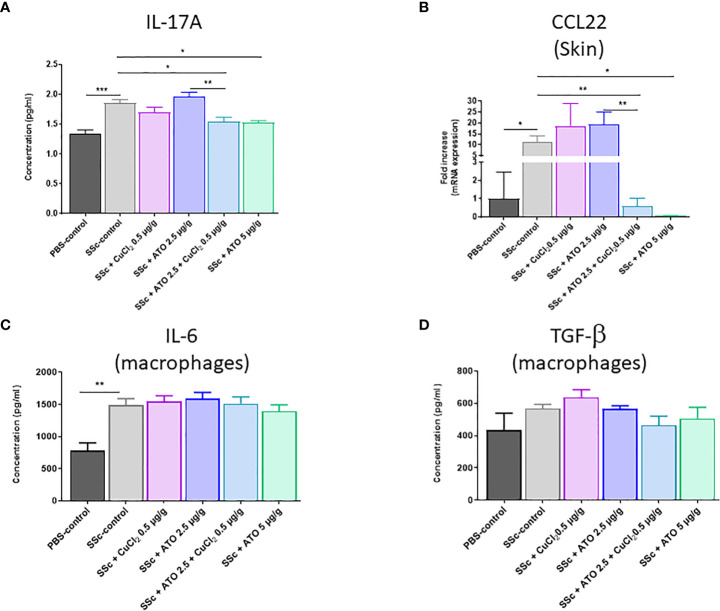
Effects of ATO 2.5 μg/g + CuCl_2_ on cytokines production. **(A)** ELISA assessment of IL-17 production by lymphocytes from the spleen after stimulation with concanavalin **(A, B)** *CCL22* mRNA expression in the skin. Data are presented as 2^(−ΔΔCT)^ relative to the levels of *β-actin*. **(C, D)** ELISA assessment of IL-6 and TGF-β production by macrophages from the spleen after stimulation with LPS. The results for each group are the mean of the measurements obtained per mouse: PBS-control (n = 5); SSc-control (n = 10); SSc-CuCl_2_ 0.5 µg/g (n = 9); SSc-ATO 2.5 µg/g (n = 9); SSc-ATO 2.5 µg/g + CuCl_2_ 0.5 µg/g (n = 10); SSc-ATO 5 µg/g (n = 10). *p <0.05; **p<0.01; ***p<0.001. *Ex vivo* measurements were realized in duplicate for each mouse.

Co-treatment with ATO 2.5 μg/g + CuCl_2_ significantly reduced by 17% the expression of IL-17A by lymphocytes compared to the HOCl-SSc-control group (p<0.05) (**Figure 5A**). The co-treatment acted similarly to a high dose of ATO on the expression of IL-17A by lymphocytes (-18%, p<0.01). In our study, we showed an increased expression of *CCL-22* in the skin of HOCl-SSc mice compared to the PBS-control group (p<0.05). This expression was reduced by the co-treatment with ATO 2.5 μg/g + CuCl_2_ and by a high concentration of ATO compared to HOCl-SSc mice (-95% p<0.01 and -99.3% p<0.05, respectively) (**Figure 5B**).

In macrophages, IL-6 production was significantly increased and TGF-β also tended to be higher in HOCl-SSc mice versus PBS-control mice but any of the treatments applied to animals in the experimental groups resulted in a modification of cytokine production compared to untreated HOCl-SSc mice (**Figures 5C, D**).

## Discussion

ATO is an inorganic salt used as a treatment for APL and is under investigation for some autoimmune diseases (16–18); however, toxic side effects are a concern (19). ATO has been shown to be the most effective single agent for the treatment of APL and, following co-treatment with ATO and all-*trans* retinoic acid, long-term survival of patients with APL has increase to 80–90% (20). An increasing number of preclinical studies have consistently shown that ATO is effective against non-cancerous disorders, especially autoimmune diseases. In the past decade, clinical data have shown that, while most side effects of ATO are mild and can include fatigue, nausea, vomiting, diarrhea, abdominal pain and peripheral neuropathy, excessive use of ATO can also induce hepatotoxicity, nephrotoxicity and cardiotoxicity (21–23). Most of these studies showed that ATO-induced organ damage is dose dependent; therefore, limiting ATO cumulative dosage would reduce these side effects. The use of ATO has recently been highlighted for the treatment of chronic autoimmune diseases. In such disorders, the acceptability of side effects is obviously lower than in more severe diseases, such as cancer. Therefore, the use of diminished amounts of ATO in treating chronic autoimmune diseases is mandatory. Our recent studies have shown that ATO can cure scleroderma in mice (7). This beneficial effect was shown to be mediated through ROS generation by ATO, which adds its effects to the constitutive production of ROS by SSc fibroblasts to selectively kill them as they contain too low levels of GSH. In the present study, we used CuCl_2_ to induce an oxidative burst through a Fenton-like reaction to potentiate the beneficial effects of ATO, thereby enabling us to reduce the ATO dosage and the risk of side effects. The co-treatment was tested in a mouse model of SSc and effectively reduced cutaneous fibrosis by inducing ROS production and cell death in activated diseased fibroblasts through a drop in the NRF2 signaling pathway. In parallel, the co-treatment altered the activation of immune cells to decrease inflammation and fibrosis, both of which are hallmarks of SSc.

With regard to the results obtained in a previous work published in Frontiers in Immunology (14), we chose the lowest copper concentration that modified the GSH concentration and the survival rate in tumor cells. This choice was also determined by the fact that we wanted to use the lowest dose to avoid toxicity in animals. Indeed, preliminary tests showed that a high dose of CuCl_2_ (4 µg/g) alone is toxic to mice (data not shown). Furthermore, a low dose of CuCl_2_, equivalent to doses used in food supplements, is sufficient to potentiate the effect of a low dose of ATO (2.5 µg/g) to the same level as that of a high dose of ATO (5 µg/g), as studied in a mouse model of cGvHD. In this study, we first observed that the co-treatment induced oxidative stress in NIH 3T3 cells and increased cell death compared to the same dosage of ATO or CuCl2 alone. Accordingly, Kavian et al., showed that ATO induced strong oxidative stress with a drop in reduced GSH levels in activated diseased fibroblasts, able to selectively kill them (7). These activated fibroblasts are a key element in the development of SSc and their depletion through an induction of sufficiently strong oxidative stress explained the decreased severity of the disease. *In vitro*, CuCl_2_ potentiated the pro-oxidative effect of ATO alone on activated fibroblasts as, in the presence of H_2_O_2_, Cu^2+^ is able to generate through a Fenton-like reaction the highly toxic radical HO. that oxidized glutathione (GSSG) (13).

We subsequently evaluated the efficacy of ATO + CuCl_2_ *in vivo*, in our mouse model of HOCl-induced SSc. It has previously been reported that ATO is able to limit the occurrence of SSc induced by repeated injections of HOCl (7). We used the same model where BALB/c mice received daily subcutaneous injections of HOCl to induce the disease. A low dose of ATO combined with CuCl_2_ decreased skin thickness and the expression of pro-fibrotic markers (*α-SMA* and *Collagen I*) in the HOCl-induced SSc mice model. The addition of CuCl_2_ potentiated the effect of ATO alone at low doses to reach an effect as powerful as that observed for a high dose of ATO. The Fenton-like Cu^2+^ divalent cation associated with ATO improved ROS production to a threshold that deleted diseased fibroblasts. These results are similar to those of previous studies by Kavian et al., who showed that ATO decreased skin fibrosis in a mouse model of chronic cGvHD and in the mouse model of SSc (7, 24). Furthermore, we recently demonstrated that CuCl_2_ potentiates the therapeutic effect of low-dose ATO alone on skin fibrosis in a mouse model of sclerodermatous cGvHD (14). In humans, an effective dose (0.16 mg/kg) can induce side effects such as skin reactions, gastrointestinal disorders and a reversible increase in transaminases, leading to discontinuation of treatment (25). The use of half the dosage of ATO is of great importance for patients who will have to be treated over a long period.

The major pathway by which cells counteract high ROS levels is through the activation of the NRF2 antioxidant pathway (26, 27). Nuclear translocation of the transcription factor NRF2 leads to the transcription of antioxidant genes in cells (28). The loss of NRF2 in cancer cells increases oxidative stress, which can result in diminished tumorigenesis and increase the sensitivity to chemotherapy (29, 30). Treating SSc animals with a high dose of ATO at 5 μg/g or with co-treatment of ATO 2.5 μg/g + CuCl_2_ 0.5 μg/g significantly reduced *NRF2* expression compared to untreated HOCl-SSc animals. Here again, addition of CuCl_2_ improved the effect of ATO low dose. To confirm this effect of ATO 2.5 μg/g + CuCl_2_ 0.5 μg/g on the NRF2 pathway, we quantified the expression of its two target genes, *NQO1* and *GCLC* (31). The expression of *NQO1* and *GCLC* was also significantly reduced by the co-treatment and by ATO high dose. As GCLC is a key enzyme for GSH synthesis and protection against oxidative stress, a drop in *NRF2* expression and its target genes explains the high sensitivity of diseased fibroblasts treated with either a high dose of ATO or with the combination of ATO and CuCl_2_ (7). Our group has already explored the role of NRF2 in scleroderma and has shown that increasing NRF2 by dimethylfumarate (DMF) prevents HOCl-induced SSc. As in cancer cells, diseased SSc fibroblasts overexpress ROS, which increases their proliferation rates (32). A return to normal can be achieved either by decreasing ROS using anti-oxidant agents, such as with DMF, or by increasing ROS beyond the viability threshold to induce apoptosis of diseased activated cells, such as with ATO high dose or in combination with CuCl_2_.

SSc is a heterogeneous autoimmune disease characterized by interconnected hallmarks represented by aberrant immune activation and by fibroblast dysfunction leading to extracellular matrix deposition and fibrosis. The immune cells are largely involved in the pathophysiology of SSc. Their uncontrolled activation contributes to maintaining the fibro-inflammatory phenomenon of the disease. We therefore studied the ATO + CuCl_2_ co-treatment’s effect on the recruitment and activation of the innate and adaptive immune system. It has been described that CD4+ T lymphocytes are abnormally activated during the development of SSc and that this exacerbated activation contributes to the pathogenesis of the disease (33). B lymphocytes are also key elements in the development of SSc. Indeed, B lymphocytes have the ability to produce cytokines to maintain the loop of inflammation and fibrosis but also to produce autoantibodies (34). Whereas a low dose of ATO alone influenced neither the number of immune cells nor their activation status, we observed that associating CuCl_2_ with ATO 2.5 μg/g lowered the percentage of B lymphocytes and also reduced the activation of CD4^+^ T lymphocytes in a similar manner to ATO high dose. These results are in agreement with those of Zhao et al., who showed that ATO is able to induce a decrease in B lymphocytes but also in T lymphocyte activation (35).

The co-treatment did not decrease the percentage and the activation of macrophages in the spleen. Only ATO high dose was able to reduce the percentage and the activation of macrophages. As well, it has been shown that chronic exposure of cells to ATO induces immunocytotoxicity on human macrophages (36).

The mouse model we used reflects the phenotype observed in SSc patients, as they present increased levels of IL-6 and TGF-β in sera and tissue (37, 38). Co-treatment with ATO and CuCl_2_ significantly decreased the level of IL-17A in the spleen compared to low dose of ATO. Cytokines such as IL-17A are produced by Th17 lymphocytes. The main role of these cells is to protect the host against pathogens. However, this protection is associated with a risk of developing immune-mediated inflammatory diseases (39). An increase in Th17 cells and IL-17 cytokine was found in peripheral blood and target organs in animal models of SSc (39). In humans, this increase is associated with skin fibrosis (40). In addition, we observed that ATO 2.5 μg/g + CuCl_2_ decreased the expression of *CCL22*, a chemokine expressed by macrophages that attracts Th17 lymphocytes *via* the CCR4 receptor. CCL22 is increased in the serum of patients with diffuse or limited SSc compared to control patients. Higher rates of CCL22 were associated with pitting scars and younger ages at onset of the pathology (41).

Finally, our results are in agreement with data from the literature, which have shown that ATO diminishes the expression of the cytokine IL-17 produced by Th17 cells but also the chemokine CCL22 expressed by macrophages (42, 43).

Altogether, these results evidence the interest of combining ATO 2.5 μg/g with CuCl_2_ 0.5 μg/g to treat SSc. The use of CuCl_2_ with ATO enabled us to treat mice with half of the amount of ATO, to obtain the same curative results. Being able to decrease the cumulative dose of any treatment is always a step forward. It helps to decrease or abrogate potential harmful effects but also avoids weakening the patients. Our data showed the important role of ROS in the effects of the combination of low-dose ATO associated with Cu^2+^ ions on overactivated fibroblasts (Graphical abstract). The association of CuCl_2_ with ATO treatment could also be effective in other autoimmune or pro-inflammatory diseases that begin with a loss of tolerance to modified self-antigens and immune system abnormalities, such as rheumatoid arthritis or systemic lupus erythematosus, where ROS are commonly used as destructive or modifying agents of cellular components that act as signaling molecules in and between immune cells.

## Data availability statement

The raw data supporting the conclusions of this article will be made available by the authors, without undue reservation.

## Ethics statement

The animal study was reviewed and approved by DAP #26065.

## Author contributions

Conceptualization, Methodology, FR, FB. Investigation, CC, MT. Formal analysis, CC, CN, MT, DR-G, FB. Writing – Original Draft, CC, CN. Writing – Review CC, CN, FR, DR-G, FB. Funding Acquisition, FR. Supervision, FB, CN. All authors contributed to the article and approved the submitted version.
