# Supplementary material for: Optimal combination of arsenic trioxide and copper ions to prevent autoimmunity in a murine HOCl-induced model of systemic sclerosis
Source: Front Immunol. 2023 Mar 30;14:1149869. doi: 10.3389/fimmu.2023.1149869 (PMC10097895; doi:10.3389/fimmu.2023.1149869)
Supplement: Supplementary file 1 [file Table_1.pdf]

SUPPLEMENTARY TABLE 1

**Murine primers used for RT-qPCR**		
Gene promoter	Forward sequence	Reverse sequence
β-actin	ACCACCATGTACCCAGGCATT	CCACACAGAGTACTTGCGCTCA
NRF2	CTGAACTCCTGGACGGGACTA	CGGTGGGTCTCCGTAAATGG
α-SMA	CTACGAACTGCCTGACGGG	GCTGTTATAGGTGGTTTCGTGG
Collagen I	TGTTCGTGGTTCTCAGGGTAG	TTGTCGTAGCAGGGTTCTTTC
CCL22	AGGTCCCTATGGTGCCAATGT	CGGCAGGATTTTGAGGTCCA
NQO1	AGGATGGGAGGTACTCGAATC	AGGCGTCCTTCCTTATATGCTA
GCLC	GGGGTGACGAGGTGGAGTA	GTTGGGGTTTGTCCTCTCCC
